# Effects of an Individualized mHealth-Based Intervention on Health Behavior Change and Cardiovascular Risk Among People With Metabolic Syndrome Based on the Behavior Change Wheel: Quasi-Experimental Study

**DOI:** 10.2196/49257

**Published:** 2023-11-29

**Authors:** Dandan Chen, Hui Zhang, Jingjie Wu, Erxu Xue, Pingping Guo, Leiwen Tang, Jing Shao, Nianqi Cui, Xiyi Wang, Liying Chen, Zhihong Ye

**Affiliations:** 1 Nursing Department Affiliated Sir Run Run Shaw Hospital Zhejiang University School of Medicine Hangzhou China; 2 Department of Nursing Guizhou Provincial People's Hospital Guiyang China; 3 Nursing Department Women's Hospital Zhejiang University School of Medicine Hangzhou China; 4 Institute of Nursing Research Zhejiang University School of Medicine Hangzhou China; 5 Institute of Nursing Research Department of Nursing of the Fourth Affiliated Hospital Zhejiang University School of Medicine Hangzhou China; 6 School of Nursing Kunming Medical University Kunming China; 7 School of Nursing Shanghai Jiao Tong University Shanghai China

**Keywords:** metabolic syndrome, health behavior, cardiovascular risk, mobile health, behavior change wheel

## Abstract

**Background:**

Metabolic syndrome (MetS) is a common public health challenge. Health-promoting behaviors such as diet and physical activity are central to preventing and controlling MetS. However, the adoption of diet and physical activity behaviors has always been challenging. An individualized mobile health (mHealth)–based intervention using the Behavior Change Wheel is promising in promoting health behavior change and reducing atherosclerotic cardiovascular disease (ASCVD) risk. However, the effects of this intervention are not well understood among people with MetS in mainland China.

**Objective:**

We aimed to evaluate the effects of the individualized mHealth-based intervention using the Behavior Change Wheel on behavior change and ASCVD risk in people with MetS.

**Methods:**

We conducted a quasi-experimental, nonrandomized study. Individuals with MetS were recruited from the health promotion center of a tertiary hospital in Zhejiang province, China. The study involved 138 adults with MetS, comprising a control group of 69 participants and an intervention group of 69 participants. All participants received health education regarding diet and physical activity. The intervention group additionally received a 12-week individualized intervention through a WeChat mini program and a telephone follow-up in the sixth week of the intervention. Primary outcomes included diet, physical activity behaviors, and ASCVD risk. Secondary outcomes included diet self-efficacy, physical activity self-efficacy, knowledge of MetS, quality of life, and the quality and efficiency of health management services. The Mann-Whitney *U* test and Wilcoxon signed rank test were primarily used for data analysis. Data analysis was conducted based on the intention-to-treat principle using SPSS (version 25.0; IBM Corp).

**Results:**

Baseline characteristics did not differ between the 2 groups. Compared with the control group, participants in the intervention group showed statistically significant improvements in diet behavior, physical activity behavior, diet self-efficacy, physical activity self-efficacy, knowledge of MetS, physical health, and mental health after a 12-week intervention (*P*=.04, *P*=.001, *P*=.04, *P*=.04, *P*=.001, *P*=.04, *P*=.04, and *P*<.05). The intervention group demonstrated a statistically significant improvement in outcomes from pre- to postintervention evaluations (*P*<.001, *P*=.03, *P*<.001, *P*=.04, *P*<.001, *P*<.001, and *P*<.001). The intervention also led to enhanced health management services and quality.

**Conclusions:**

The individualized mHealth-based intervention using the Behavior Change Wheel was effective in promoting diet and physical activity behaviors in patients with MetS. Nurses and other health care professionals may incorporate the intervention into their health promotion programs.

## Introduction

### Background

Metabolic syndrome (MetS) is a leading global public health challenge [1]. It is characterized by increased waist circumference (WC), elevated systolic or diastolic blood pressure, elevated triglyceride levels, reduced high-density lipoprotein cholesterol (HDL-C) levels, and elevated fasting plasma glucose readings [2]. The global prevalence of MetS is estimated to be approximately 25%. In 2018, over a billion people worldwide were affected by MetS [3]. The prevalence of MetS in the United States and Iran is approximately 22.9% and 34.7%, respectively [4,5]. According to the China Nutrition and Health Surveillance (2015-2017), the prevalence of MetS was 31.1% among adults aged ≥20 years [1]. As MetS comprises established risk factors for atherosclerotic cardiovascular disease (ASCVD) and type 2 diabetes mellitus (T2DM), it is expected that individuals with MetS will have a 2-fold increased risk of developing ASCVD and a 5-fold increased risk of developing type 2 diabetes mellitus [6]. In addition, MetS is positively associated with other noncommunicable diseases, such as cancer and premature mortality [7,8]. The cost associated with MetS is in trillions, and it is expected to increase in the future [9]. In Germany, Spain, and Italy, the economic burden of MetS on health services was €24,427 (US $25,834); €1900 (US $2009.60); and €4877 (US $5158.40) million, respectively [10]. To reduce the burden of the disease, it is important to design effective interventions to manage MetS.

### The Need for Individualized mHealth-Based Intervention

An international panel recommendation and guideline showed that a healthy lifestyle is the first-line intervention for MetS prevention and management [11,12]. It is challenging to identify the lifestyle that is individually most important [13]. Previous research has demonstrated that relatively simple lifestyle intervention programs emphasizing sufficient physical activity and avoiding excess saturated fats, salt, and simple sugars can improve all components of MetS [14,15] and are also the focus of current health behavior interventions for people with MetS [16]. However, most of these interventions were delivered face-to-face or by telephone [17,18] and required great commitment from patients and health care professionals owing to their manpower consumption, economic costs, time consumption, and lack of immediate results. This greatly limited the implementational scalability of interventions in promoting metabolic health. To overcome the drawbacks of traditional lifestyle interventions, mobile health (mHealth) was considered as a cost-effective way to deliver interventions. Previous studies have reported that mHealth-based interventions promote physical activity and positive lifestyle changes in individuals with MetS [19,20]. However, these mHealth-based interventions, originating abroad or from Hong Kong, may not be suitable for patients with MetS in mainland China.

In addition, most mHealth-based interventions offer general “one-size-fits-all” lifestyle education for people with MetS [19,21], which has achieved limited outcomes [22]. The concept of individualization refers to creating intervention content based on an individual’s specific characteristics, such as existing behaviors, stages of behavior change, preferences, and barriers [23]. A previous study confirmed that individualized lifestyle education helped patients with MetS incorporate lifestyle interventions into their daily lives [24]. According to a meta-analysis, individualized interventions may be an effective approach for adults to reduce MetS parameters [23], which could be attributed to the fact that they meet patients’ individualized needs and provide tailored measures, considering that every patient is unique and has distinct requirements. Despite the potential of mHealth-based interventions to provide individualized recommendations, the current functionality limitations of mHealth media, including email, websites, mobile phones, and SMS text messaging, restrict the extent to which individualized recommendations can be offered in response to the diverse needs of patients [19,21]. A WeChat mini program grounded in WeChat has diversified functions, supports developers to implement individualized functions, and can provide customized significant and meaningful experiences according to the needs of patients. It does not need to log in to a specific website [25] and avoids the limitations of a long development cycle, high cost, and troublesome installation of mobile apps [26,27]. Hence, in this case, a WeChat mini program provides solutions for meeting the diversified individual needs of patients with MetS.

### The Need for Theory in mHealth-Based Intervention

**Figure 1:**
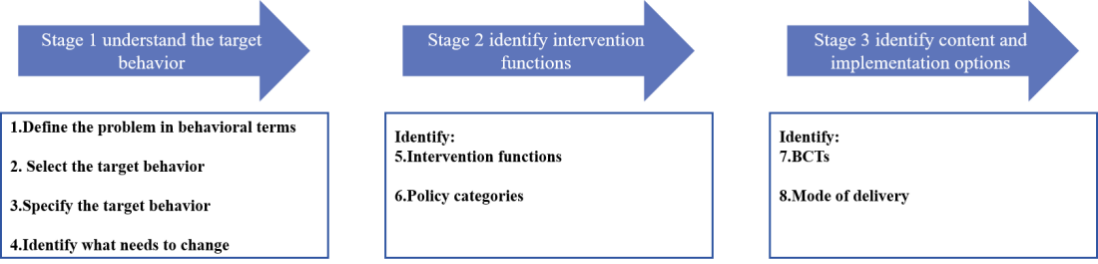
Stages involved in an intervention development using the Behavior Change Wheel [31] (used with permission from authors). BCT: behavior change technique.

It was observed that most mHealth-based interventions for patients with MetS tend to be poor at applying theories [28,29] and have limited emphasis on evidence-based content, which may limit their success and lead to suboptimal adherence [30]. Behavioral change has multiple theories and models, but most of them ignore the background in which the target behavior occurs, pay little attention to the reflective process, have a static structure, and fail to clarify how to change the behavior [31]. Existing frameworks for behavioral change interventions also lack comprehensiveness, coherence, and behavior change models [32]. To address the limitations of the abovementioned theories and frameworks, the Behavior Change Wheel (BCW) framework was created containing 19 behavior change frameworks (Figure 1) [31]. Given the advantages of the BCW, our research team developed an individualized mHealth-based intervention program based on this theory [21,30,33-35].

### Aims of This Research

In this context, we aimed to evaluate the effects of an individualized mHealth-based intervention based on a WeChat mini program using the BCW on diet and physical activity behaviors and ASCVD risk compared with conventional face-to-face health education, providing a more effective approach to promoting positive behavior change and reducing ASCVD risk among people with MetS in mainland China.

## Methods

### Study Design

The study was a quasi-experimental, non–randomized controlled trial (RCT) and was reported according to the Transparent Reporting of Evaluations with Nonrandomized Designs Statement Checklist [36]. A quasi-experimental design was chosen because the WeChat mini program is easily accessible to everyone. To prevent contamination between the intervention and control groups, the WeChat mini program was released after a 12-week observation period in the control group, and patients with MetS were recruited to the intervention group. Therefore, we chose to conduct a quasi-experimental study and not a full RCT.

### Participants

From May to November 2022, patients with MetS were recruited from the health promotion center of a tertiary hospital in Zhejiang province, China.

The inclusion criteria were as follows: (1) patients who met the diagnostic standard of MetS proposed by the 2009 Joint Scientific Statement, including abdominal obesity–WC (≥85 cm in men; ≥80 cm in women), triglyceride (≥1.7 mmoL/L or treatment), plasma HDL-C (＜1.0 mmoL/L in men; ＜1.3 mmoL/L in women or treatment), blood pressure (systolic ≥130 and diastolic ≥85 mm Hg or treatment), and fasting plasma glucose (≥5.6 mmoL/L or treatment) [2]; (2) individuals between the ages of 18 and 65 years; (3) proficiency in using smartphones and WeChat mini programs; (4) participants who have not participated in any other MetS intervention project; and (5) those who voluntarily agreed to participate and signed informed consent forms.

The exclusion criteria were as follows: (1) patients with severe heart, liver, or kidney dysfunction, cognitive impairment, or psychiatric history; (2) those with hearing and visual impairments; (3) pregnant or lactating women; (4) those undergoing specific diet and physical activity therapy; and (5) individuals with limited physical activity due to conditions such as stroke, arrhythmia, or severe rheumatism.

### Sample Size

For this study, the sample size calculated on the basis of the total physical activity score was greater than that calculated on the basis of the dietary score in a pilot study, so the sample size was determined based on the physical activity score, which was calculated using the physical activity subscale of the Chinese Health Promoting Lifestyle Profile II, Revise [37]. We calculated that each group needed at least 57 individuals using the formula n1=n2=2(t_α_+t_β_)^2^S^2^/δ^2^, for an effect size of 0.6 to achieve 80% power and a significance level of 0.05. Considering a potential attrition rate of 20%, we aimed to recruit 138 participants, with 69 individuals assigned to each intervention and control group.

### Procedure

The patients were screened based on the eligibility criteria and were included in the study. Patients interested in participating in the study were provided a patient information sheet and consent form. Patients in the control group were recruited first with their consent form, and after a 12-week observation period, patients in the intervention group were recruited.

### Blinding

Implementing blinding for both health care professionals and participants involved in the intervention was not feasible in this study. Blinding was implemented for personnel involved in the data collection and analysis processes to minimize bias.

### Intervention

Before the intervention, patients in the control and intervention groups received health education about diet and physical activity during their examination day at the health promotion center, which lasted 30 to 40 minutes. The content focused on specific diet and physical activity recommendations for patients with MetS, mainly including the type and amount of diet and the type, frequency, and intensity of physical activity. We selected patients who were hospitalized for physical examinations, which provided ample time for them to receive health education. The intervention group additionally received a 12-week individualized intervention through a WeChat mini program and telephone follow-up in the sixth week of the intervention. Specifically, the WeChat mini program was the primary delivery mode for the intervention. Our research team developed the individualized intervention through an intensive literature review [21], following clinical guidelines [11,12,34], considering the patients’ needs through semistructured interviews and a cross-sectional survey based on the BCW [30,33], conducting experts’ consultation, and performing the development [35] and usability evaluation of the WeChat mini program. The details of the BCW applied to the intervention are presented in Multimedia Appendix 1. The specific details of the individualized interventions are outlined subsequently.

The intervention team consisted of a chief general practitioner, general educational nurse, physician in the rehabilitation department, head nurse in the cardiology department, dietitian, nurse in the endocrinology department, and 2 nursing doctoral candidates. The team members stayed connected with each other through WeChat to discuss and solve problems and to report the progress of the intervention.

**Figure 2:**
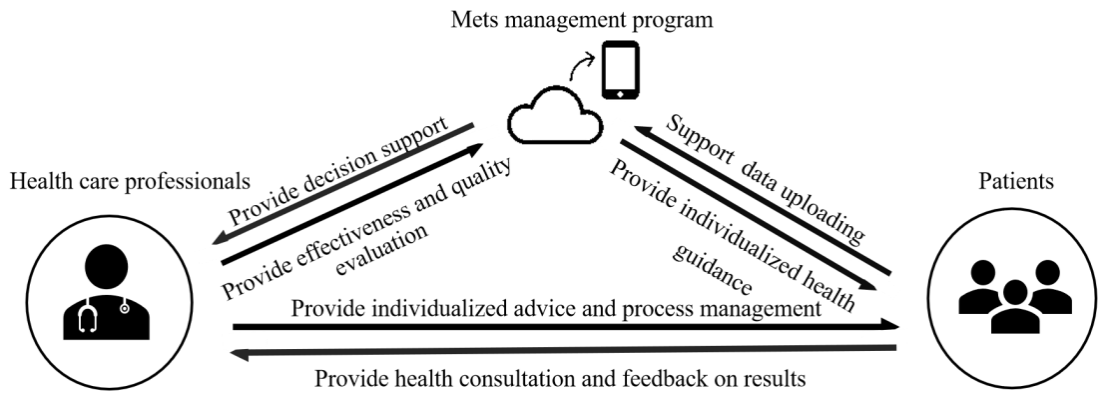
The application of the WeChat mini intervention. MetS: metabolic syndrome.

On the day of the health examination, our team member guided patients to use the WeChat mini program named “Metabolic Syndrome Management Assistant” and provided detailed answers to any questions from patients, ensuring patient familiarity with the program. The WeChat mini program includes 10 modules: (1) “Health assessment” primarily provides individualized diet and physical activity recommendations; (2) “Daily record” records patients’ physical activity and diet on a daily basis; (3) “Last 7 days report” module provides physical activity and dieting reports for the past 7 days in the form of a chart; (4) “Suggestions for behavior barriers” provide individualized solutions to obstacles to performing behaviors; (5) “Knowledge encyclopedia” introduces diagnosis, etiology, clinical manifestations, complications, physical activity guidance, dietary guidance, and other textual and video information about MetS; (6) “Health consultation and reminders” provides individualized feedback according to patients’ questions and provides physical activity and diet reminders; (7) “Questionnaires” assists users in completing web-based assessments, including health promotion lifestyle, knowledge of MetS prevention and treatment, self-efficacy of health behaviors, and quality of life; (8) “Operation guide” introduces the rules for using the program; (9) “Disclaimer” introduces the conditions under which the program is exempt from liability; and (10) “Privacy statement” introduces the content of the users’ privacy protection. Participants were encouraged to use the program at least 5 times a week [38,39]. For participants who did not log in for 2 consecutive days, the health care professional reminded them and asked for reasons. While using it, the program automatically provided patients with individualized health management recommendations, personalized behavioral barrier resolution recommendations, and personalized feedback on the type and amount of diet and the type, frequency, and intensity of physical activity based on their own uploaded data, choices, and records. The program automatically provided knowledge about MetS and health outcome assessments for patients. In addition, our research team provided behavior reminders and professional support and assistance for patients through the program. The intervention period lasted 12 weeks. Multimedia Appendix 2 shows screenshots of the WeChat mini program. The specific application of the WeChat mini program is shown in Figure 2.

The WeChat mini program was used as the primary intervention delivery method. However, according to experts’ suggestions, relying solely on the program is insufficient to promote health behavior change due to the different initiative levels of patients. Moreover, referring to previous studies [19,21], this study conducted a telephone follow-up in the sixth week of the intervention to ensure that all patients received health education. All the contents of the telephone follow-up were determined by our prior study [30]. Specifically, the health care professionals provided patients with knowledge of healthy diets. They also highlighted the importance of reasonable family dietary arrangements to ensure a balanced nutritional intake. Furthermore, they held detailed consultations with patients, exploring different approaches to guaranteeing balanced nutrition. They evaluated patients’ diet, physical activity, and goal achievement and provided individualized guidance and encouragement based on their feedback. In addition, they offered professional support and assistance in addressing any questions or confusion from patients.

### Study Outcomes

A doctoral candidate collected data from the health promotion center. The demographic and clinical characteristics were assessed at baseline. All outcomes were measured at baseline and after the intervention. The primary outcomes were diet behaviors and physical activity behaviors. Secondary outcomes included 10-year risks of ASCVD, diet self-efficacy, physical activity self-efficacy, knowledge of MetS, and quality of life.

### Primary Outcomes

Wen-jun et al [37] developed the Chinese Health Promoting Lifestyle Profile II, Revise as an assessment tool for individuals’ health-promoting behaviors [37]. The scale consists of 40 items comprising 6 dimensions: nutrition, physical activity, health responsibility, stress management, interpersonal relationships, and spiritual growth. For this study, the nutrition dimension (6 items) and physical activity dimension (8 items) of the scale were used. Each item had 4 response options, ranging from 1 (never) to 4 (routinely), resulting in total scores ranging from 6 to 24 for the diet dimension and from 8 to 32 for the physical activity dimension. A higher score indicates a healthier lifestyle choice. The psychometric properties of this scale have been found to be acceptable [30].

### Secondary Outcomes

#### 10-Year Risks of ASCVD

The Prediction for ASCVD Risk in China model is used to evaluate an individual’s risk of developing ASCVD in the next 10 years [40]. The model comprehensively considers the risk factors involved in previous prediction models, such as age, systolic blood pressure, use of antihypertensive drugs, total cholesterol, HDL-C, smoking, and diabetes, as well as the characteristics of China’s actual situation and disease spectrum. It includes WC, north-south geography, urban-rural status, and family history of ASCVD and analyzes the interaction between age and other risk factors. The Prediction for ASCVD Risk in China model is an effective tool for individual 10-year ASCVD risk assessment.

#### Health Behavior Self-Efficacy

Becker et al [41] developed the Health Behavior Self-Efficacy Scale in 1993, which comprises 28 items, is divided into 4 subscales (exercise, nutrition, health practice responsibility, and good mental state) and uses a 5-point Likert scoring method (0=almost not certain, 1=somewhat certain, 2=moderate confidence, 3=high confidence, and 4=absolute confidence) [41]. The total score ranges from 0 to 112, with higher scores indicating greater health behavior self-efficacy. This study assessed the health behavior self-efficacy of patients with MetS by selecting a nutrition self-efficacy subscale and an exercise self-efficacy subscale with a total of 14 items, and each subscale had a Cronbach α value of .806 and .912, respectively.

#### Knowledge of MetS

The Knowledge of Metabolic Syndrome Scale was developed to assess the knowledge of patients with MetS in 2010. The scale consists of three dimensions and a total of 10 items: (1) definition and diagnosis of MetS (5 items), (2) complications of MetS (2 items), and (3) prevention of MetS (3 items). Each item has 5 answer options, and the scoring is based on the number of correct answers, with a score of 10 given for each correct answer [42]. The scale’s total score ranges from 0 to 100, with a higher score indicating better knowledge of MetS. This scale has been validated and found to have acceptable reliability among Chinese populations with MetS, with a Cronbach α value of .70 [30].

#### Quality of Life

This study used the 12-item Short Form Health Survey Version 2 to measure the quality of life of patients with MetS [43,44]. This scale covers 8 domains: general health, physical functioning, role limitations due to physical problems, bodily pain, vitality, social functioning, emotional role limitations, and mental health. The physical health score can be calculated based on scores of general health, physical functioning, role limitations due to physical problems, and bodily pain domains, whereas the mental health score can be calculated based on scores of vitality, social functioning, emotional role limitations, and mental health domains. The scores for physical health and mental health were transformed into standardized scores, ranging from 0 to 100, respectively. A score of 50 or above indicates normal function, with higher scores indicating better quality of life. The Cronbach α value of the 12-item Short Form Health Survey Version 2 was .725 in this study.

#### Quality and Efficiency of Health Management Services

In line with the literature [45], we used a self-designed questionnaire to assess the quality and efficiency of health management services. The questionnaire comprised 6 items and adopted a 3-level rating system to score the responses. The possible total scores ranged from 5 to 15, with higher scores indicating greater perceived effectiveness and quality of health management services.

#### Quality Control

Our team member guided the intervention group to search for “Metabolic Syndrome Management Assistant” on WeChat and provided detailed instructions on how to use the WeChat mini program to ensure that patients could master the methods of using the program. In addition, when entering data, 2 members of our research team were required to double-enter and cross-check to ensure the accuracy of data entry. When conducting the data analysis, other team members who did not collect data performed the analysis to reduce subjective bias.

### Statistical Analysis

For categorical data, frequency and percentage were used for description. For continuous data, mean and SD were used when the distribution was normal, whereas median and quartiles were used when the data did not conform to a normal distribution. Intergroup comparisons were performed using an independent samples 2-tailed *t* test for continuous variables with a normal distribution, the Mann-Whitney *U* test for continuous variables that did not conform to a normal distribution, and the chi-square test or Fisher exact test for categorical variables. Within-group comparisons were performed using either the paired-sample *t* test (for continuous variables with a normal distribution of differences) or Wilcoxon signed rank test (for continuous variables with a nonnormal distribution of differences). Data analyses were conducted based on the intention-to-treat principle using SPSS (version 25.0; IBM Corp). A *P* value <.05 was considered statistically significant.

### Ethical Considerations

This study followed the Declaration of Helsinki and was approved by the Hospital Institutional Review Board (number 20210220-32). This study was registered with the Chinese Clinical Trial Registry (ChiCTR2100043877). Informed consent was obtained from all participants, and all data were guaranteed to be confidential. Participants were informed of their right to voluntarily withdraw from the study. All participants with MetS underwent free blood biochemical examinations for participation in our study.

## Results

### Patient Flow and Baseline Characteristics

**Figure 3:**
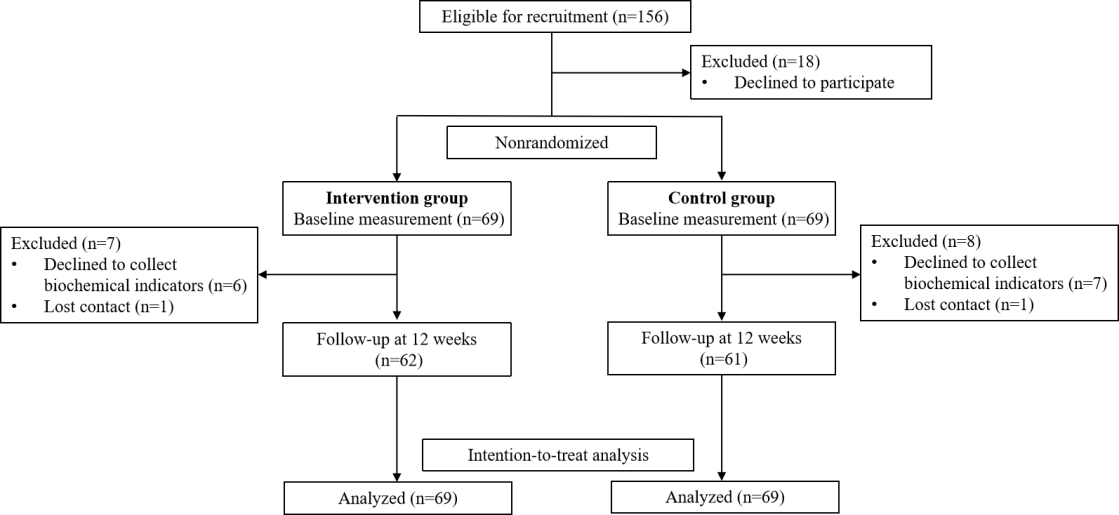
Participants’ flow.

This study included 138 adults with MetS. The intervention group comprised 69 patients; 6 patients refused data collection, and 1 patient withdrew voluntarily, resulting in a final sample of 62 patients with a dropout rate of 10.1%. Similarly, a total of 69 patients were recruited for the control group. During the 12-week observation period, 7 patients refused data collection and 1 patient withdrew voluntarily, resulting in a final sample of 61 patients with a dropout rate of 11.2%. The flowchart of the study is shown in Figure 3.

Of the 138 participants, the average age was 51.50 (range 45.75-56.00) years. Most participants were male (111/138, 80.4%), lived in cities (86/138, 62.3%), had no religious affiliation (81/138, 58.7%), had junior high school education (37/138, 26.8%), and were married (134/138, 97.1%). The detailed demographic and clinical characteristics are shown in Table 1. Overall, the 2 groups were balanced in terms of their demographic and clinical characteristics.

**Table 1:** Demographic and clinical characteristics for patients with metabolic syndrome.

Variables			Total (n=138)		Control group (n=69)		Intervention group (n=69)		Statistics			*P* value	
Age (years)			51.50 (45.75-56.00)^a^		51.13 (7.05)^b^		51 (44-55)^a^		−1.166^c^			.24	
**Sex, n (%)**										0.046^d^			.83
	Male	111 (80.4)		56 （81）		55 （80）							
	Female	27 (19.6)		13 （19）		14 （20）							
**Residence, n (%)**										2.069^d^			.36
	City	86 (62.3)		39 （57）		47 （68）							
	Town	32 (23.2)		19 （28）		13 （19）							
	Countryside	20 (14.5)		11 （16）		9 （13）							
**Region, n (%)**										0.030^d^			.86
	Yes	57 (41.3)		28 （41）		29 （42）							
	No	81 (58.7)		41 （59）		40 （58）							
**Education level, n (%)**										2.313^e^			.83
	Elementary	9 (6.5)		3 （4.3）		6 （9）							
	Junior	37 (26.8)		20 （29）		17 （25）							
	Senior	33 (23.9)		15 （22）		18 （26）							
	College	24 (17.4)		12 （17）		12 （17）							
	Undergraduate	30 (21.7)		17 （25）		13 （19）							
	Graduate or higher	5 (3.6)		2 （3）		3 （4）							
**Marital status, n (%)**										N/A^f^			.62
	Unmarried	4 (2.9)		1 （1）		3 （4）							
	Married	134 (97.1)		68 （99）		66 （96）							
**Occupation, n (%)**										7.207^e^			.53
	Leaders in government agencies enterprises and institutions	39 (28.3)		19 （28）		20 （29）							
	Staff	13 (9.4)		7 （10）		6 （9）							
	Business and service industry personnel	23 (16.7)		14 （20）		9 （13）							
	Worker	14 (10.1)		4 （6）		10 （15）							
	Farmer	4 (2.9)		2 （3）		2 （3）							
	Freelancer	31 (22.5)		17 （25）		14 （20）							
	Unemployed	4 (3)		3 （4）		1 （1）							
	Retirees	8 (5.8)		3 （4）		5 （7）							
	Other	2 (1.4)		0 （0）		2 （3）							
**Average monthly household income (€; US $), n (%)**										3.350^e^			.90
	<1000 (136.7)	2 (1.4)		1 （1）		1 （1）							
	1000-2000 (273.4)	3 (2.2)		2 （3）		1 （1）							
	2000-4000 (546.8)	7 (5.1)		3 （4）		4 （6）							
	4000-6000 (820.1)	11 (8)		4 （6）		7 （10）							
	6000-8000 (1093.5)	13 (9.4)		5 （7）		8 （12）							
	8000-10,000 (1366.9)	20 (14.5)		12 （17）		8 （12）							
	10,000-15,000 (2050.4)	29 (21)		14 （20）		15 （22）							
	>15,000 (2050.4)	53 (38.4)		28 （41）		25 （36）							
**Family history of chronic diseases**										0.116^d^			.73
	Yes	70 (50.7)		34 （49）		36 （52）							
	No	68 (49.3)		35 （51）		33 （48）							
**Undergone metabolic surgery**										N/A			<.001
	Yes	3 (2.2)		2 （3）		1 （1）							
	No	135 (97.8)		67 （97）		68 （99）							
**Take antihypertensive medication**										0.000^d^			<.001
	Yes	50 (36.2)		25 （36）		25 （36）							
	No	88 (63.8)		44 （64）		44 （64）							
**Take antidiabetic medication**										0.549^d^			.46
	Yes	19 (13.8)		11 （16）		8 （12）							
	No	119 (86.2)		58 （84）		61 （88）							
**Take lipid-lowering medication**										0.673^d^			.42
	Yes	15 (10.9)		9 （13）		6 （9）							
	No	123 (89.1)		60 （87）		63 （91）							
**Number of metabolic parameters**										0.340^d^			.84
	Three	82 (59.4)		40 （58）		42 （61）							
	Four	45 (32.6)		24 （35）		21 （30）							
	Five	11 (8)		5 （7）		6 （9）							

^a^Values are presented as mean (range).

^b^Values are presented as mean (SD).

^c^Mann-Whitney *U* test.

^d^Chi-square test.

^e^Fisher exact test.

^f^N/A: not applicable.

### Effects of the Individualized mHealth-Based Intervention on Health Behaviors, ASCVD Risk, Health Behavior Self-Efficacy, Knowledge of MetS, and Quality of Life

The primary outcomes were diet behavior, physical activity behavior, and ASCVD risk. The secondary outcomes included diet self-efficacy, physical activity behavior, knowledge of MetS, and quality of life. As shown in Table 2, no substantially differences were found between the 2 groups before the individualized mHealth-based intervention. According to the intergroup comparison analysis, participants showed statistically significant improvements in diet behavior, physical activity behavior, diet self-efficacy, physical activity self-efficacy, knowledge of MetS, physical health, and mental health after the individualized mHealth-based intervention (*P*=.04, *P*=.001, *P*=.04, *P*=.04, *P*=.001, *P*=.04, and *P*=.04). However, ASCVD risk was not statistically significantly improved after the intervention (Table 3). In addition to comparing the results between the 2 groups, we also compared the results within each group. According to the findings of the within-group comparison, the results showed no statistically significant differences in outcomes between the control group in the pre- and postintervention assessments (Table 4). However, the intervention group demonstrated statistically significant improvements in outcomes from pre- to postintervention evaluations (*P*<.001, *P*=.03, *P*<.001, *P*=.04, *P*<.001, *P*<.001, and *P*<.001), except for the ASCVD risk (Table 5). No adverse events occurred during the intervention period.

**Table 2:** Comparison of primary and secondary outcomes among the intervention group versus the control care group before the intervention.

Outcomes	Control group (n=69)	Intervention group (n=69)	Statistics	*P* value
Diet behavior, median (range)	18.000 (17.000-21.500)	18.000 (16.000-20.000)	−1.733^a^	.08
Physical activity behavior, mean (SD)	17.145 (5.160)	16.348 (4.331)	0.983^b^	.33
ASCVD^c^ risk, median (range)	0.037 (0.027-0.055)	0.048 (0.032-0.048)	−1.163^a^	.25
Diet self-efficacy	19.000 (15.000-21.500)^d^	17.942 (4.459)^e^	−0.604^a^	.55
Physical activity self-efficacy, median (range)	14.000 (10.500-20.500)	16.000 (11.500-21.000)	−0.817^a^	.41
Knowledge of metabolic syndrome, median (range)	4.000 (1.500-5.000)	4.000 (3.000-5.500)	−1.921^a^	.06
Physical health, median (range)	50.000 (37.500-62.500)	51.250 (38.750-57.500)	−0.403^a^	.69
Mental health, median (range)	56.250 (45.625-64.375)	57.500 (47.500-66.250)	−0.634^a^	.53

^a^Mann-Whitney *U* test.

^b^Independent samples *t* test.

^c^ASCVD: atherosclerotic cardiovascular disease.

^d^Values are presented as mean (range).

^e^Values are presented as mean (SD).

**Table 3:** Comparison of primary and secondary outcomes among the intervention group versus the control group after the intervention.

Outcomes	Control group (n=69)	Intervention group (n=69)	Statistics	*P* value
Diet behavior, median (range)	18.000 (17.000-21.500)	20.000 (18.000-22.500)	−2.045^a^	*.04^b^*
Physical activity behavior, mean (SD)	17.478 (4.934)	20.435 (4.885)	−3.573^c^	*.001*
ASCVD^d^ risk, median (range)	0.034 (0.024-0.043)	0.044 (0.026-0.060)	−1.51^a^	.13
Diet self-efficacy, median (range)	19.000 (15.500-21.500)^e^	20.029 (4.162)^f^	−2.050^a^	*.04*
Physical activity self-efficacy, median (range)	14.000 (10.500-20.000)	17.000 (13.000-20.000)	−2.028^a^	*.04*
Knowledge of metabolic syndrome, median (range)	4.000 (2.000-5.000)	5.000 (3.000-6.000)	−3.428^a^	*.001*
Physical health, median (range)	50.500 (39.250-62.375)	55.500 (49.250-65.875)	−2.012^a^	*.04*
Mental health, median (range)	58.750 (48.750-65.625)	58.750 (55.375-77.500)	−2.102^a^	*.04*

^a^Mann-Whitney *U* test.

^b^Italicization means significant effects at *P*<.05.

^c^Independent samples *t* test.

^d^ASCVD: atherosclerotic cardiovascular disease.

^e^Values are presented as mean (range).

^f^Values are presented as mean (SD).

**Table 4:** Comparison of outcomes before and after the intervention in the control group.

Outcomes	Before the intervention (n=69)	After the intervention (n=69)	Statistics	*P* value
Diet behavior, median (range)	18.000 (17.000-21.500)	18.000 (17.000-21.500)	−1.930^a^	.05
Physical activity behavior, median (range)	16.000 (14.000-21.500)	17.000 (14.000-20.000)	−1.138^a^	.26
ASCVD^b^ risk, median (range)	0.037 (0.027-0.055)	0.034 (0.024-0.043)	−1.489^a^	.14
Diet self-efficacy, median (range)	19.000 (15.000-21.500)	19.000 (15.500-21.500)	−1.459^a^	.15
Physical activity self-efficacy, median (range)	14.000 (10.500-20.500)	14.000 (10.500-20.000)	−0.688^a^	.50
Knowledge of metabolic syndrome, median (range)	4.000 (1.500-5.000)	4.000 (2.000-5.000)	−1.935^a^	.06
Physical health, mean (SD)	50.453 (14.473)	51.391 (14.327)	−1.733^c^	.09
Mental health, mean (SD)	54.928 (13.204)	58.290 (13.856)	−0.937^c^	.35

^a^Wilcoxon signed rank test.

^b^ASCVD: atherosclerotic cardiovascular disease.

^c^Paired samples *t* test.

**Table 5:** Comparison of outcomes before and after the intervention in the intervention group.

Outcomes	Before the intervention (n=69)	After the intervention (n=69)	Statistics	*P* value
Diet behavior, median (range)	18.000 （16-20）	20.000 （18-22.500）	−5.598^a^	<*.001^b^*
Physical activity behavior, mean (SD)	16.348 (4.331)	20.435 (4.885)	−2.309^c^	*.03*
ASCVD^d^ risk, median (range)	0.048 (0.032-0.048)	0.044 (0.026-0.060)	−0.679^a^	.50
Diet self-efficacy, mean (SD)	17.942 (4.459)	20.029 (4.162)	−7.262^c^	<*.001*
Physical activity self-efficacy, median (range)	16.000 (11.500-20.000)	17.000 (13.000-20.000)	−2.065^a^	*.04*
Knowledge of metabolic syndrome, median (range)	4.000 (3.000-5.500)	5.000 (3.000-6.000)	−5.769^a^	<*.001*
Physical health, mean (range)	51.250 (38.750-57.500)	55.500 (49.250-65.875)	−5.792^a^	<*.001*
Mental health, median (range)	57.50 (47.500-66.250)	58.75 (55.375-77.500)	−3.643^a^	<*.001*

^a^Wilcoxon signed rank test.

^b^Italicization means significant effects at *P*<.05.

^c^Paired samples *t* test.

^d^ASCVD: atherosclerotic cardiovascular disease.

### Evaluation of the Quality and Efficiency of Health Management Services

After the intervention, a questionnaire survey of 6 health care professionals participating in the individualized mHealth-based intervention was conducted. According to our survey, 83% (5/6) of health care professionals believed that the individualized mHealth-based intervention saved time; 67% (4/6) found that the intervention reduced substantial economic investment, and 100% (6/6) of them reported a significant reduction in manpower. In addition, 67% (4/6) of health care professionals found that the intervention provided much support to patients and greatly relieved their work pressure. Finally, 100% (6/6) of them thought that the intervention improved their relationships with the patients (Table 6).

**Table 6:** Evaluation of the quality and efficiency of health management services (n=6).

Items			Values, n (%)
**Amount of time saved**			
	None	0 (0)	
	Some	1 (17)	
	Much	5 (83)	
**Extent of economic input reduction**			
	None	1 (17)	
	Some	1 (17)	
	Much	4 (67)	
**Extent of manpower input reduction**			
	None	0 (0)	
	Some	0 (0)	
	Much	6 (100)	
**Amount of assistance provided to patients**			
	None	0 (0)	
	Some	2 (33)	
	Much	4 (67)	
**Extent of relief from work pressure**			
	None	0 (0)	
	Some	2 (33)	
	Much	4 (67)	
**Extent of improvement in the health care professional-patient relationship**			
	None	0 (0)	
	Some	3 (50)	
	Much	3 (50)	

## Discussion

### Principal Findings

This is the first study to assess the effects of an individualized mHealth-based intervention using the BCW on health behavior change and ASCVD risk among patients with MetS in mainland China. This study indicates that individualized mHealth-based interventions resulted in considerable improvements in diet and physical activity behaviors, diet and physical activity self-efficacy, knowledge of MetS, and quality of life. In addition, the intervention also enhanced the quality and efficiency of the health management services. However, the ASCVD risk did not show a statistically significant difference at 3 months.

This mHealth-based intervention was built upon our prior research [21,30,33-35], which included a WeChat mini program and a telephone follow-up, with a primary focus on the WeChat mini program. The results of our study showed that after an individualized mHealth-based intervention, diet and physical activity behaviors improved in patients with MetS, which was consistent with previous findings. Lihua [46] conducted a 6-month network platform–based comprehensive intervention among patients with MetS and found that the proportion of patients with a reasonable diet and regular exercise increased after the intervention compared with before the intervention. Wong et al [20] found that a mobile app–based lifestyle intervention was more effective in maintaining exercise compared with booklet support among patients with MetS. A study involving 7 European countries found that providing individualized nutrition advice through a web-based intervention was more effective in promoting dietary behavior change than conventional methods [47]. In our study, the main reasons for the improvement in health behaviors were that the WeChat mini program provides a solution for providing individualized care services to patients, which helps improve patients’ health behaviors [48]. Specifically, the program can provide individualized diet and physical activity recommendations for patients with MetS based on their biochemical indicators and work life types. The program can record health behaviors and automatically provide feedback based on patients’ daily and weekly records. To address barriers to adopting healthy behaviors, the program provides individualized suggestions according to patients’ options. At the same time, the program can provide individualized health consultations for patients. These individualized measures contribute to enhancing patients’ adherence to healthy behaviors.

ASCVD is one of the main complications of MetS and the main target for preventing and treating MetS [49,50]. A nurse-led lifestyle intervention program showed that the 10-year risk of cardiovascular disease did not statistically significantly decrease during the 3-month intervention period among patients with MetS [18], which was similar to our study. This may be related to the short intervention period (12 weeks). It has been observed that studies reporting positive findings on ASCVD risk typically used a study period of 5 months, 1 year, or 2 years [51-54]. Future studies are needed to evaluate the effects of the intervention over 12 weeks on ASCVD risk. In addition, there are many factors that can affect ASCVD risk, including family history, total cholesterol, and tobacco use [40]. Therefore, more comprehensive consideration is required when interpreting the findings.

Our research determined that the individualized mHealth-based intervention was an effective approach for enhancing health behavior self-efficacy, which aligned with previous research. The app-based intervention group showed an improvement in exercise self-efficacy from baseline to 24 weeks [20]. Zheng et al [18] found that statistically significant improvements in health behavior self-efficacy were observed when patients followed a lifestyle intervention program consisting of 30- to 40-minute face-to-face health education sessions, reading health education manuals, and 6 follow-up telephone consultations. Our study attributes the effects of the intervention to some contributing factors. Health care professionals can help patients resolve queries and provide encouragement toward continued healthy behaviors through the WeChat mini program and a telephone follow-up. Moreover, the program presents knowledge and skills related to diet and physical activity in videos, pictures, and texts, which may be attractive for patients with MetS to learn. These measures enhanced patients’ confidence in performing health behaviors, statistically significantly improving their health behavior self-efficacy.

The results of this study showed that, after the individualized mHealth-based intervention, the intervention group demonstrated an increase in knowledge of the MetS score compared with the control group. The baseline survey of this study found that patients lacked knowledge of MetS. A contributing factor is that MetS is largely asymptomatic [55], and many individuals with this condition may fail to perceive its harm, resulting in a lack of preventive measures. Therefore, this study embedded specific content on the diagnosis, etiology, complications, diet, and physical activity recommendations for MetS into the WeChat mini program to enhance patients’ knowledge of MetS.

This study found that the individualized mHealth-based intervention statistically significantly improved the quality of life of patients with MetS. This result corresponded to a meta-analysis that stated that lifestyle interventions statistically significantly improve health-related quality of life in all domains [56]. Numerous studies have indicated that physical activity exerted a positive effect not only on human physiological functions but also on emotion regulation [57,58]. Studies have confirmed that a healthy diet can also help alleviate negative emotions such as anxiety and depression [59-61]. The above research provides potential explanations for the observed improvements in the quality of life among individuals with MetS.

This study revealed that health care professionals perceived an enhancement in the quality and efficiency of health management services following the individualized mHealth-based intervention. Dongxing et al [62] developed a precision health management platform based on the WeChat mini program. The platform improved both the quality and efficiency of hospital health management services, which was in accordance with the findings of our study. The improvements in this study may be mainly attributed to the program’s automation of the delivery of individualized diet and physical activity recommendations, providing feedback on progress toward achieving goals and offering suggestions for overcoming behavioral barriers. These features reduced repetitive workloads, eased work intensity, and enhanced work efficiency for health care professionals.

### Clinical and Research Implication

For patients with MetS, our study used a relatively convenient, inexpensive, and innovative technology that allowed more participants to easily access the intervention through WeChat, making it more accessible to patients. The mHealth-based intervention with more individualized elements provided patients with individualized care and resulted in larger and more appropriate changes in health behaviors compared with routine health education. In addition, the mHealth-based intervention encouraged patients to change from passive participation in health management to active participation, which was conducive to strengthening their awareness of proactive health maintenance. For health care professionals, the individualized mHealth-based behavior intervention requires less manpower and time investment, making it a more efficient and cost-effective solution. This has resulted in the improved quality and efficiency of health management services. Therefore, our study provides a new perspective for promoting patients’ health behavior change and improving the quality and efficiency of health management.

### Limitations

This study has some limitations. A limitation of our study is that it was not an RCT. However, it prevented contamination caused by accessibility of the WeChat mini program to everyone. A standardized RCT design is necessary to further validate the effects of the intervention. Meanwhile, the data used in this study were obtained from a tertiary hospital, which may have introduced sampling bias despite meeting the statistical requirements. Hence, the generalizations of the research findings should be interpreted with caution. In the future, multicenter studies with a large sample size should be conducted. Furthermore, this study did not use an objective tool to measure physical activity and evaluate the quality and efficiency of health management services because of limited costs and time. The data collected were self-reported, which may have introduced the potential for recall bias. Finally, due to time and resource limitations, this study conducted a 12-week health behavior intervention and did not follow-up on the patients’ health behavior maintenance status after the intervention, which may weaken the empirical validity of the research results.

### Future Studies

In future studies, the “Metabolic Syndrome Management Assistant” needs further improvement in terms of appearance, knowledge presentation form, and functions of existing modules. The research team will continue to collaborate with software engineers to optimize and upgrade the program based on patients’ feedback. Our research team will also evaluate the medium- and long-term effects of the intervention through large-scale clinical randomized controlled trials and explore its effectiveness and economic cost-effectiveness. In addition, future research will integrate artificial intelligence technology to deliver more precise and intelligent care to patients with MetS, for example, by providing precise prediction, intelligent question answering, and intelligent recommendation for patients.

### Conclusions

In conclusion, the mHealth-based behavior intervention with individualized assessments, strategies, feedback, and suggestion components and using the BCW, including a WeChat mini program and a telephone follow-up, effectively increased patients’ dietary behavior, physical activity behavior, diet self-efficacy, physical activity self-efficacy, MetS knowledge, quality of life, and quality and efficiency of health management services. Nurses and other health care professionals may incorporate the intervention into their health promotion programs. Future studies will optimize and upgrade the WeChat mini program using artificial intelligence technology to provide more personalized and intelligent services. Studies with larger samples and a longer follow-up period are also needed to evaluate the effect of the individualized mHealth-based behavior intervention on behavior change and ASCVD risk among people with MetS.

## Appendix

Multimedia Appendix 1 The details of how the Behavior Change Wheel is applied.

Multimedia Appendix 2 The screenshots of the WeChat mini program.

## Abbreviations

ASCVD: atherosclerotic cardiovascular disease
BCW: Behavior Change Wheel
HDL-C: high-density lipoprotein cholesterol
RCT: randomized controlled trial
WC: waist circumference

## Data Availability

Data are available from the first author or corresponding author on reasonable request.
