# Supplementary figures and images for: Effects of an Individualized mHealth-Based Intervention on Health Behavior Change and Cardiovascular Risk Among People With Metabolic Syndrome Based on the Behavior Change Wheel: Quasi-Experimental Study
Source: J Med Internet Res. 2023 Nov 29;25:e49257. doi: 10.2196/49257 (PMC10720605; doi:10.2196/49257)

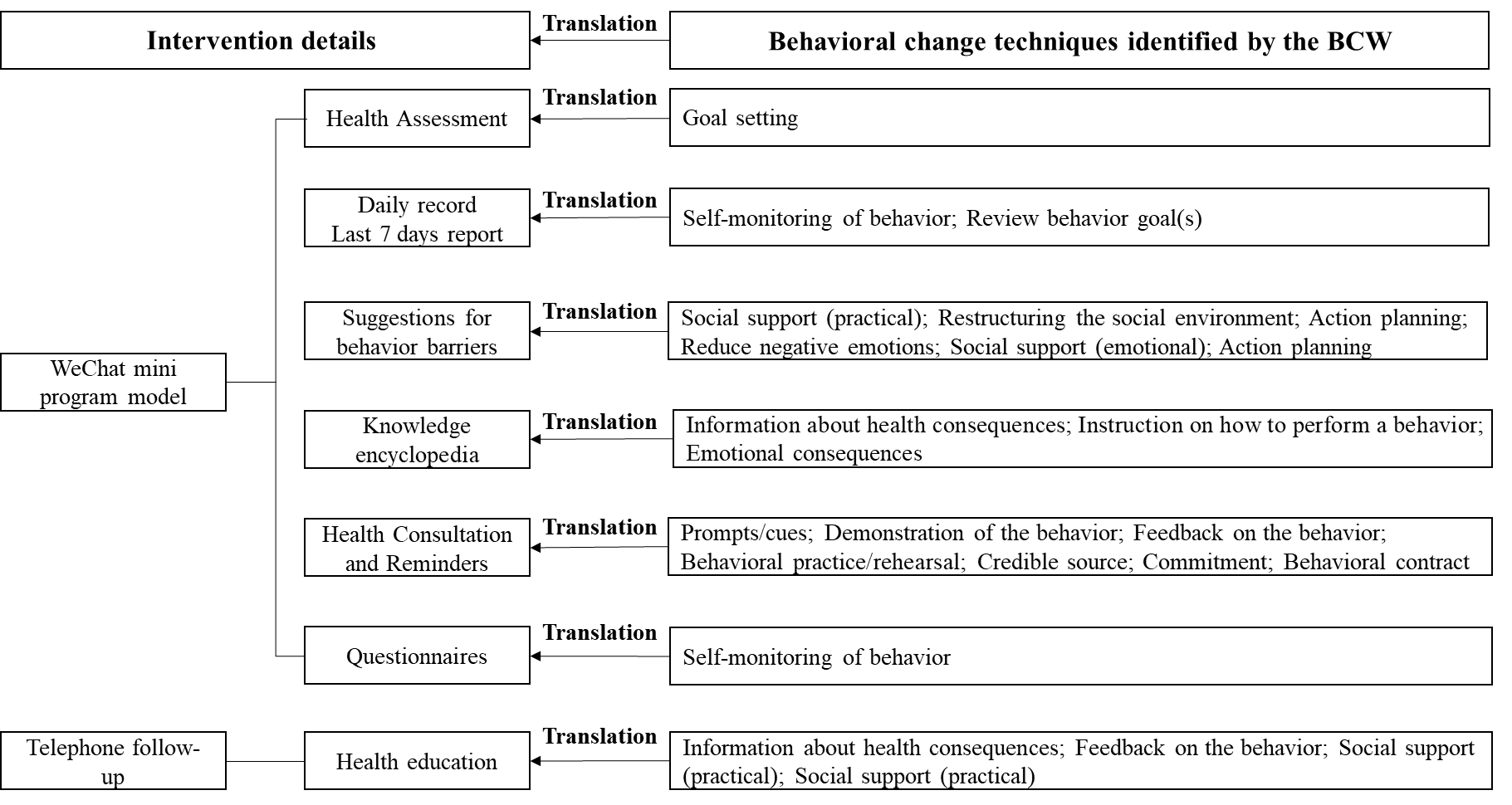
Figure S1. The details of the BCW applied in the intervention.

Supplement: Multimedia Appendix 1 [file jmir_v25i1e49257_app1.docx]
