# Supplementary material for: Effects of an Individualized mHealth-Based Intervention on Health Behavior Change and Cardiovascular Risk Among People With Metabolic Syndrome Based on the Behavior Change Wheel: Quasi-Experimental Study
Source: J Med Internet Res. 2023 Nov 29;25:e49257. doi: 10.2196/49257 (PMC10720605; doi:10.2196/49257)
Supplement: Multimedia Appendix 2 [file jmir_v25i1e49257_app2.docx]

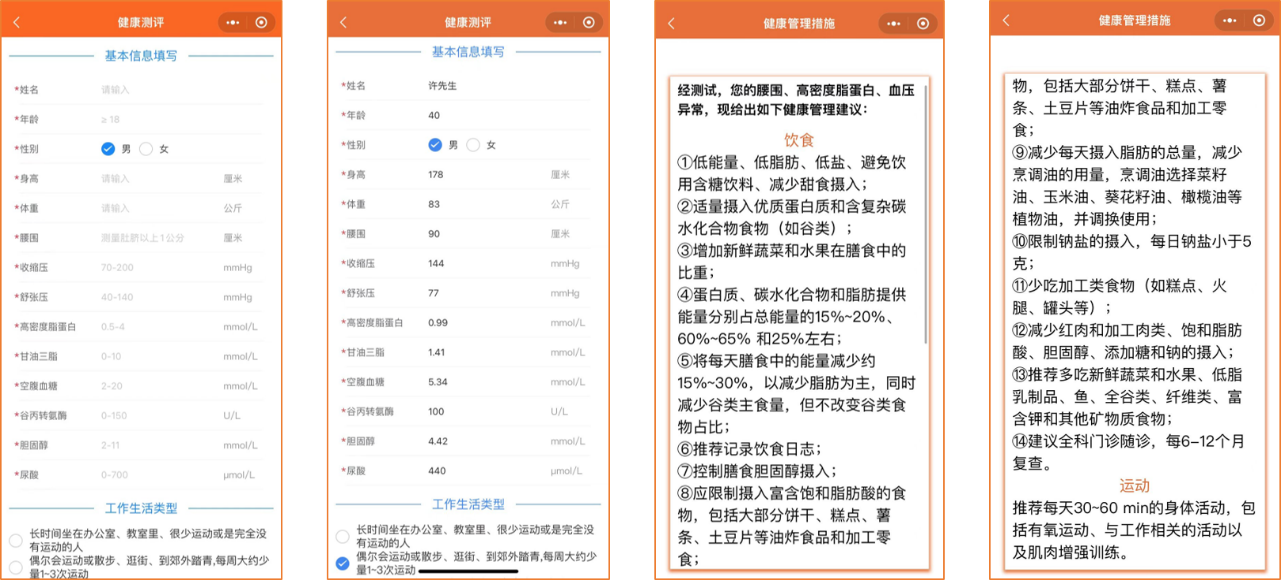


Figure S1. The “Health Assessment” interface.


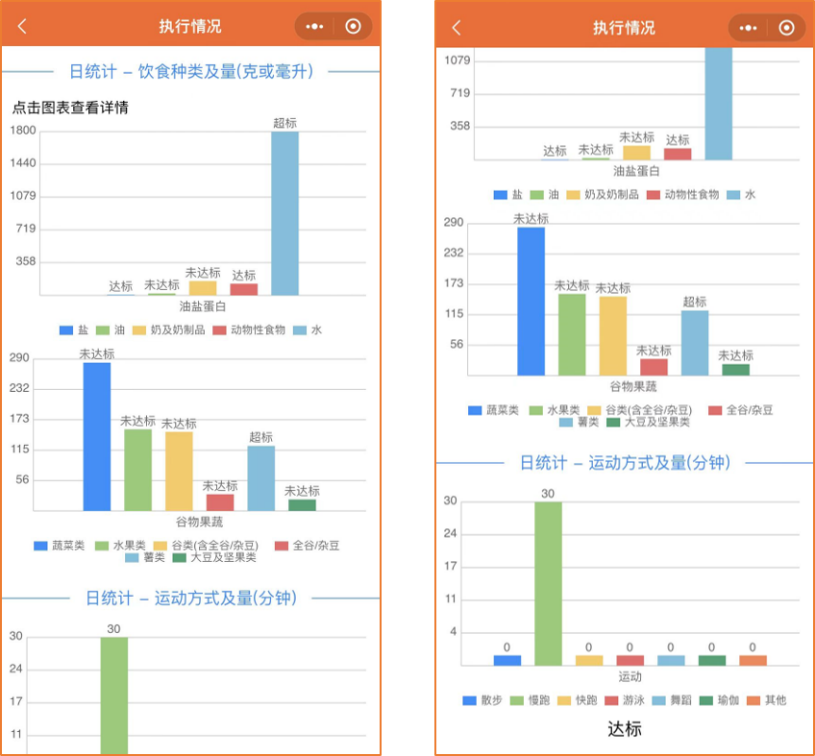


Figure S2. The “Daily Records” interface.


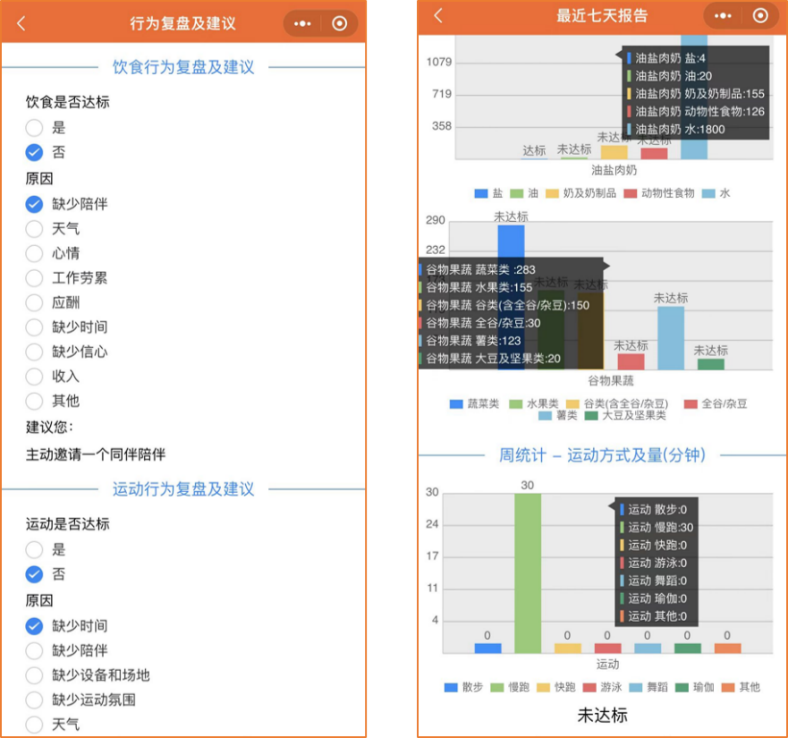


Figure S3. The “Suggestions for Behavior Barriers” interface and “Recent Seven-Day Report” interface.


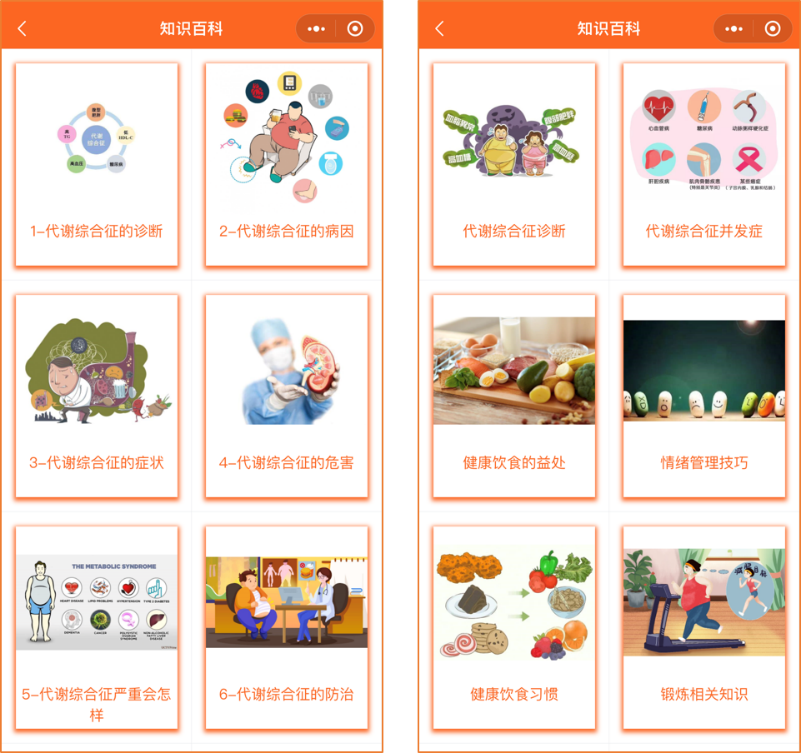


Figure S4. The “Knowledge Encyclopaedia” interface.


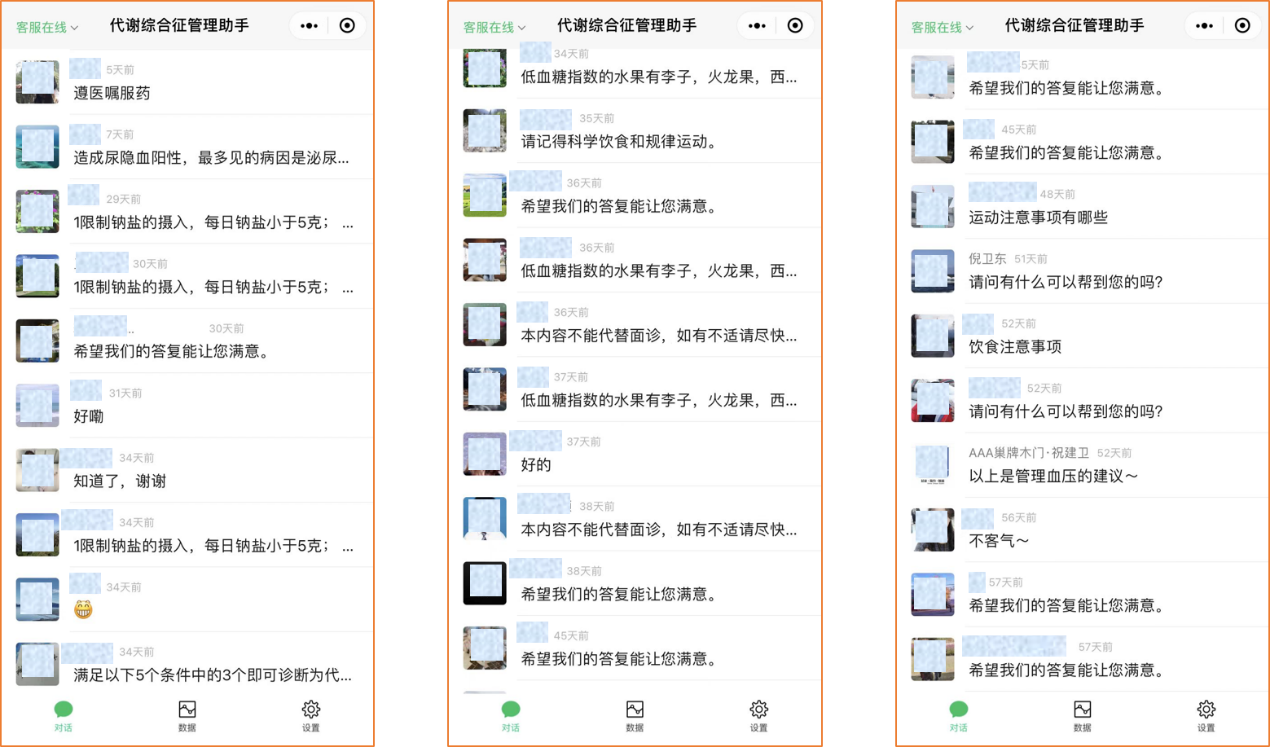


Figure S5. The “Health consultation and reminders” interface.


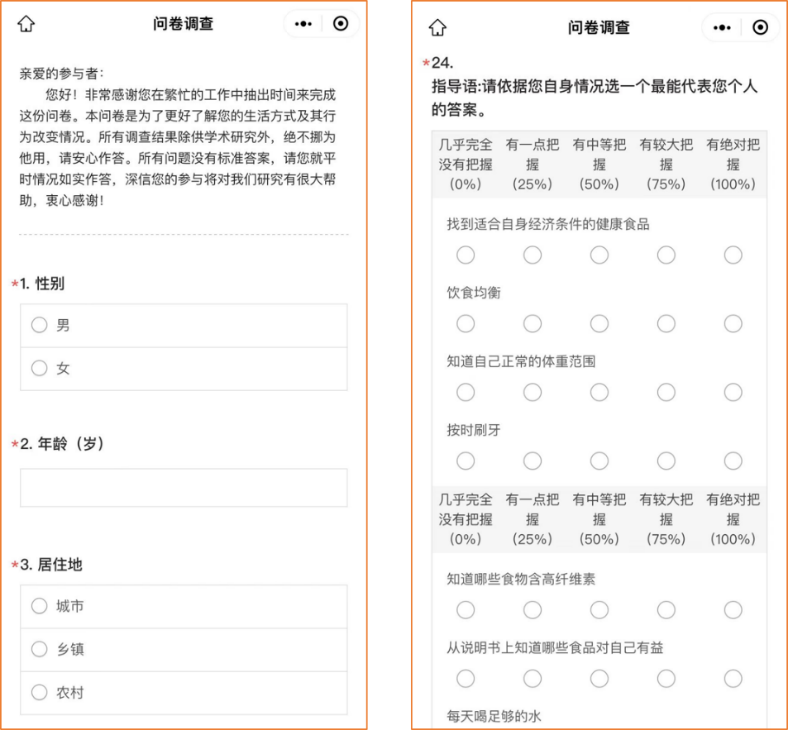


Figure S6. The “Questionnaire survey” interface.
